# SEEG-guided therapeutic decision-making in drug-resistant epilepsy: retrospective analysis and clinical framework

**DOI:** 10.1007/s00701-026-06910-7

**Published:** 2026-06-12

**Authors:** Marta Codes, Alvaro Bedoya, Abel Ferres, Albert Mas, Mar Carreño, Maria Centeno, Estefania Conde, Antonio Donaire, Marta Olivera, Eugenia Pujol-Ayach, Lorena Gomez, Jordi Rumia, Pedro Roldan

**Affiliations:** 1 Institut Clínic de Neurociències (ICN), Department of Neurosurgery, Hospital Clínic of Barcelona, University of Barcelona, C/Villarroel 170, 08036 Barcelona, Spain; 2 Institut Clínic de Neurociències (ICN), Department of Neurology, Hospital Clínic of Barcelona, University of Barcelona, Barcelona, Spain; 3 Institut Clínic de Neurociències (ICN), Department of Anesthesiology and Resuscitation, Hospital Clínic of Barcelona, University of Barcelona, Barcelona, Spain

**Keywords:** Drug-resistant epilepsy, Stereoelectroencephalography, Clinical decision-making, Radiofrequency thermocoagulation, Epilepsy surgery, Epileptogenic zone

## Abstract

**Background:**

Stereoelectroencephalography (SEEG) plays a central role in the presurgical evaluation of patients with drug-resistant epilepsy, particularly when noninvasive investigations are inconclusive. Beyond localization of the epileptogenic zone (EZ), its role in guiding therapeutic decision-making remains incompletely defined.

**Objective:**

To evaluate the safety, clinical outcomes, and therapeutic impact of SEEG, including SEEG-guided radiofrequency thermocoagulation (RF-TC), and to develop a structured clinical decision-making framework based on SEEG findings.

**Methods:**

We performed a retrospective analysis of 71 consecutive patients with drug-resistant epilepsy who underwent SEEG monitoring between 2016 and 2025. Clinical, neuroimaging, and electrophysiological data were analyzed, along with procedural variables, complications, and outcomes following RF-TC and resective surgery. SEEG findings were used to stratify patients into distinct therapeutic pathways.

**Results:**

A total of 958 electrodes were implanted (mean 14 ± 4.5 per patient). SEEG confirmed the preimplantation hypothesis in 85% of cases. Based on SEEG findings, 52% of patients were candidates for resective surgery, while 48% were managed conservatively or with neuromodulation strategies. RF-TC was performed in 37 patients (52%), resulting in seizure improvement in 73% at 12 months, although complete seizure freedom was limi. Response to RF-TC influenced subsequent treatment decisions, favoring conservative management in responders and resective surgery in non-responders. Favorable outcomes (Engel I–II) after resective surgery were achieved in 71% of patients at 12 months. Complications were predominantly minor, with a 1% rate of permanent neurological deficit.

**Conclusions:**

SEEG is a safe and effective tool not only for localizing the EZ but also for structuring therapeutic decision-making in drug-resistant epilepsy. SEEG findings enable patient stratification into individualized treatment pathways and support a dynamic, staged approach in which RF-TC may serve as both a therapeutic and decision-modulating tool. This framework highlights the evolving role of SEEG as a central platform in modern epilepsy surgery.

## Introduction

The need to offer alternative treatments to patients with drug-resistant epilepsy has led to the appearance of pre-surgical evaluation techniques that allow the better localization of the epileptogenic areas to assess the possibility of performing surgery with curative intent.

Despite the continuous improvement and refinement of non-invasive techniques, which typically constitute the initial phase of the investigation, there is a considerable number of patients for whom the results of these tests are not conclusive enough to guide a clear decision, and invasive investigations are necessary [8, 20, 36].

Indications for invasive evaluation may differ among epilepsy centers, but SEEG is generally accepted when precise localization of the EZ is required to plan accurate surgical resection or when functional mapping is necessary to ensure surgical safety [20, 36].

While the role of SEEG in localizing the epileptogenic zone is well established, its integration as a structured decision-making tool within the presurgical pathway remains less clearly defined. In particular, there is limited evidence describing how SEEG findings systematically influence the selection between resective surgery, minimally invasive approaches such as radiofrequency thermocoagulation (RF-TC), neuromodulation strategies, or the decision to avoid surgery altogether.

In this context, the present study aims to evaluate not only the safety and clinical outcomes of SEEG, but also its role in guiding therapeutic decision-making. Based on our experience, we propose a clinically applicable SEEG-guided decision-making framework derived from real-world practice.

Rather than focusing solely on implantation safety or postsurgical seizure outcomes, this series emphasizes the real-world clinical role of SEEG as a hypothesis-driven diagnostic tool that directly informs both surgical and non-surgical decision-making.

Finally, an important and underreported contribution of this series is the analysis of patients in whom SEEG findings led to the decision not to proceed with resective surgery. In this cohort, SEEG played a critical role in identifying multifocal epilepsy, overlap with eloquent cortex, or insufficiently localized epileptogenic networks, thereby preventing ineffective or unsafe surgical interventions. In this context, SEEG should be viewed not only as a gateway to surgery, but also as a decisive tool for avoiding inappropriate surgical treatment [2, 4, 6, 7, 9, 11, 12, 14, 19, 22–24, 26, 27, 30–33].

Based on these considerations, we evaluated the role of SEEG as a dynamic tool for individualized therapeutic decision-making in real-world clinical practice.

## Methods

We analyzed all patients with drug-resistant epilepsy who underwent SEEG monitoring as part of a presurgical evaluation at our center between 2016 and 2025. Patients with generalized and multifocal epilepsy syndromes were excluded from the analysis, as the study focused on candidates for resective epilepsy surgery. For non-resective cases, alternative neuromodulation-based treatments, such as deep brain stimulation or vagus nerve stimulation, were considered according to institutional protocols.

A comprehensive descriptive analysis was performed to define the basal characteristics of the enrolled patients, encompassing the results of previous non-invasive tests, findings from the SEEG monitoring, results of thermocoagulation if performed, and any associated complications related to the technique. The surgical results of those who underwent resection surgery for EZ were also analyzed.

Collected variables included demographic characteristics, epilepsy history, imaging findings, number and laterality of implanted electrodes, monitoring duration, hospital stay, SEEG-related complications, RF-TC outcomes, and postoperative seizure outcomes after resective surgery.

**Fig. 1:**
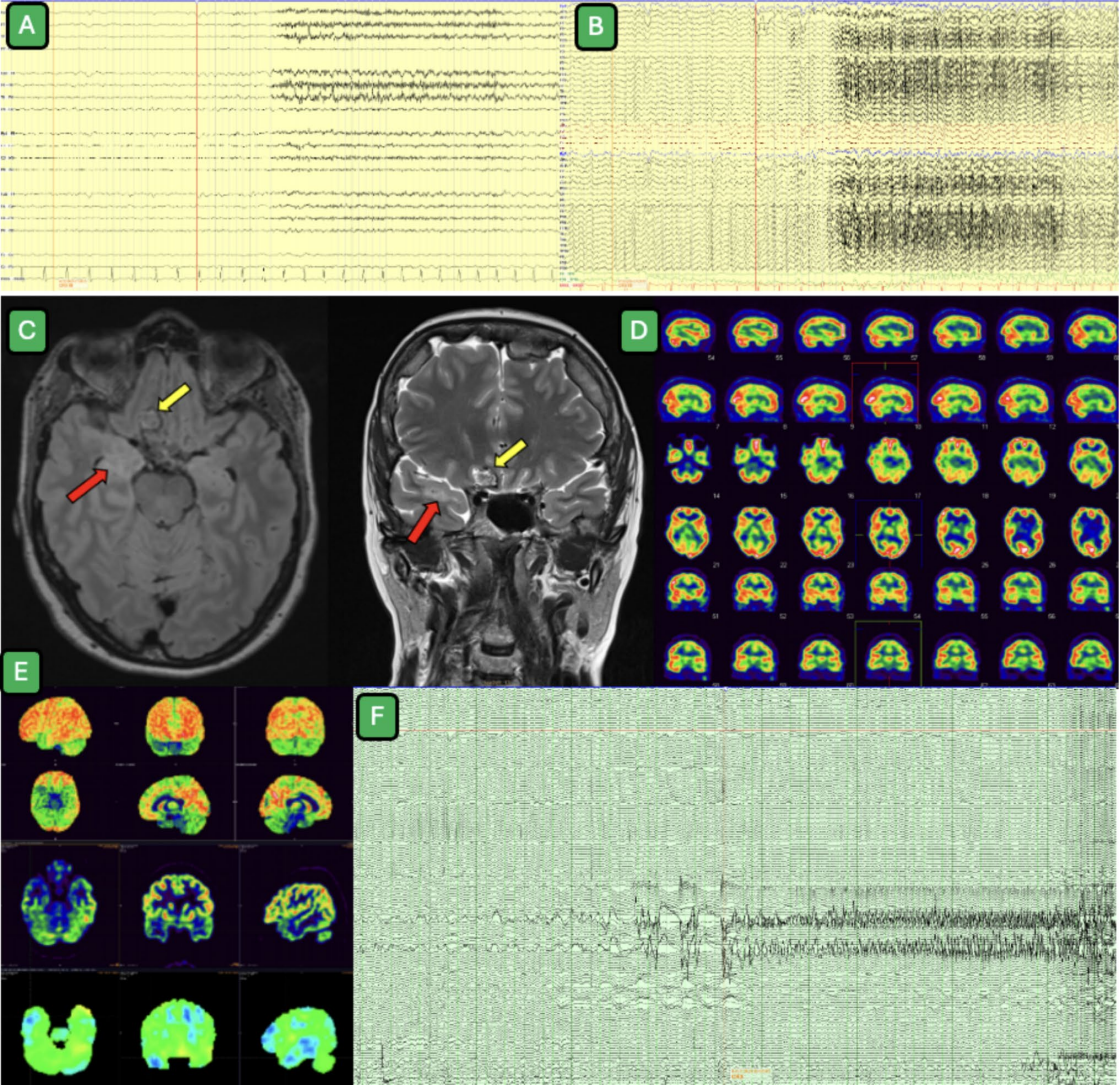
Case of a patient with drug-resistant epilepsy and dual pathology. **A** Bipolar EEG montage showing rhythmic spiculated activity in the right frontotemporal region. **B** EEG during seizure onset with progression in the right temporal chain. **C** MRI demonstrating right temporal sclerosis (red arrow) and right frontobasal cavernoma (yellow arrow). **D** SPECT with hyper uptake in the right temporal pole. **E** PET showing hypometabolism in right temporal and anterobasal frontal regions. **F** SEEG confirming seizure onset in hippocampal contacts O1-2 and H1-2, leading to ipsilateral temporal lobectomy via an endoscopic transorbital approach

Prior to selection for SEEG, all patients underwent an extensive non-invasive presurgical evaluation within a multidisciplinary epilepsy surgery team including specialists in neurology, neurosurgery, neuropsychology, nuclear medicine, and neuroradiology. This evaluation comprised a detailed clinical history, surface video-electroencephalographic (EEG) recordings, and structural and functional neuroimaging studies, including contrast-enhanced magnetic resonance imaging (MRI), thin-slice contrast-enhanced cranial computed tomography (CT), single-photon emission computed tomography (SPECT), and/or positron emission tomography (PET). Comprehensive neuropsychological assessments were also performed to evaluate higher cortical and cognitive functions (Fig. 1).

The indications for SEEG at our center included MRI-negative epilepsy, discordance between electroclinical and neuroimaging findings, the presence of multiple or discordant lesions, and overlap of the presumed EZ with eloquent cortical areas.

Hypothesis confirmation was defined as concordance between the preimplantation electroclinical hypothesis and the seizure onset zone identified by SEEG recordings.

Based on this hypothesis-driven approach, we structured a reproducible SEEG-guided decision-making framework as follows.

### Operational SEEG-guided decision-making framework

To enhance reproducibility and clinical applicability, we propose an operational SEEG-guided decision-making framework based on four sequential steps derived from our institutional practice:

- Step 1: Hypothesis validation
- SEEG recordings are used to confirm or refute the preimplantation electroclinical hypothesis. Concordance between non-invasive data and SEEG-defined seizure onset zone (SOZ) supports progression toward therapeutic intervention.
- Step 2: Epileptogenic zone characterization
- he EZ is classified according to:
  - Focal versus multifocal organization
  - Spatial extent of the epileptogenic network
  - Relationship with eloquent cortical areas
- This step determines the feasibility and safety of resective strategies.
- Step 3: Functional and therapeutic testing (RF-TC)
- In selected cases, SEEG-guided RF-TC is performed to:
  - Evaluate the functional relevance of the identified EZ
  - Assess the potential clinical impact of focal lesioning
  - Provide preliminary therapeutic benefit
- Patients are stratified as responders or non-responders based on clinical and electrographic outcomes.
- Step 4: Therapeutic decision pathway
  - Resective surgery (well-localized EZ, acceptable functional risk, poor RF-TC response)
  - Conservative or delayed strategy (significant RF-TC response or non-disabling seizures)
  - Neuromodulation (multifocal epilepsy or high surgical risk)
  - No surgical intervention (patient refusal or insufficient clinical indication)

This structured framework reflects a dynamic and individualized approach, in which SEEG acts as both a diagnostic and decision-modulating platform.

Electrode implantation was performed using frameless stereotactic neuronavigation with intraoperative imaging guidance. Since 2023, procedures have been performed using the Neuromate® robotic system (Renishaw) [28].

Postoperative imaging was systematically obtained to exclude procedure-related complications.

RF-TC was performed through implanted electrodes after completion of SEEG monitoring in selected patients approved by the multidisciplinary epilepsy committee. Thermocoagulation targets were defined according to ictal onset patterns and stimulation mapping while avoiding eloquent cortical regions.

The procedure was carried out using a dedicated radiofrequency generator (F.L. Fischer®, Neuro N50, Germany). RF-TC was selectively applied to electrode contacts located within the EZ, as defined by ictal onset patterns and corroborated by electrical stimulation mapping, ensuring that targeted contacts did not involve eloquent cortical regions.

After completion of the monitoring period and RF-TC, electrodes were removed under general anesthesia. A postoperative CT scan was performed to confirm the absence of bleeding. Electroclinical data were reviewed to accurately characterize the EZ, and findings were discussed at the institutional Epilepsy Committee to guide multidisciplinary therapeutic decision-making. Follow-up of all included patients has been maintained to date, and outcomes of those who subsequently underwent EZ resective surgery were analyzed, including type of resection, procedure-related complications, and postoperative seizure outcomes.

## Results

We analyzed all patients with drug-resistant epilepsy who underwent EEG monitoring via SEEG electrodes in our center from 2016 to 2025, with a total of 71 consecutive patients (Table 1).

**Table 1:** Clinical and surgical characteristics of 71 patients undergoing SEEG (2016–2025)

Clinical and surgical characteristics		
Category	Variable	Result
Population	Included patients	71
	Sex	33 males (46%), 38 females (54%)
	Mean age at SEEG	35.4 ± 12.1 years
	Mean epilepsy onset age	15 ± 10.9 years
Pre-surgical evaluation	Lesional epilepsy	50 (70%)
	Non-lesional epilepsy	21 (30%)
	Multiple lesions	9 (18% of lesional)
	Most frequent lesion type	Cortical dysplasia 37 (64%), tumors 9 (15%), postsurgical gliosis 7 (12%), heterotopia 5 (9%)
Time intervals	Diagnosis → Epilepsy Committee	18.7 ± 13.4 years
	Decision for SEEG → Implantation	19 ± 32.6 months
Surgical aspects	Total electrodes	958
	Mean electrodes per patient	14 ± 4.5
	Unilateral implantation	38 patients (54%)
	Bilateral implantation	33 patients (46%)
	Mean surgical duration	200 ± 55.8 min (≈3 h)
Monitoring & hospital stay	Monitoring duration	13.7 ± 4.6 days
	Hospital stay	16.8 ± 5 days
Complications (SEEG)	Minor radiological findings without clinical impact	
	- Small Intracerebral hemorrhage - Small Subarachnoid hemorrhage	8 (11%) 3 (4%)
	Clinically significant complication	
	- Infection - Symptomatic hemorrhage requiring surgery	2 (3%) 1 (1%)
	Permanent deficit	1 (1%)
	Mortality	0
Thermocoagulation complications (*n* = 5, 14%)	Hemiparesis	2 (5%)
	Aphasia/dysphasia	2 (5%)
	Dysnomia	1 (3%)
Non-surgical patients (*n* = 34, 48%)	Rejection of surgery	9 (26%)
	Multifocal epilepsy	8 (24%)
	Partial seizure improvement with pharmacological adjustment + RF-TC	7 (20%)
	High surgical risk (EZ located within eloquent brain areas)	6 (18%)
	Absence of disabling seizures	4 (12%)

### Patient demographic features

A total of 71 patients underwent stereotactic procedures for intracerebral electrode implantation in our center. There were 33 males (46%) and 38 women (54%), whose mean age at the time of the SEEG examination was 35.4 ± 12.1 years and the mean age of onset of epilepsy was 15 ± 10.9 years.

### Pre-surgical evaluations

Imaging pre-surgical tests using MRI classified the epilepsy type as lesional 50 patients (70%), or non-lesional 21 patients (30%). In those 50 patients with lesional epilepsy, 9 of them (18%) had multiple lesions in the MRI.

The most frequently observed lesions were cortical dysplasia in 37 cases (64%), followed by tumoral lesions in 9 cases (15%).

Other described lesions included gliosis associated with previous surgeries in 7 cases (12%) and heterotopias in 5 cases (9%).

### Time intervals

The mean time between diagnosis of epilepsy and presentation of the case in the Epilepsy Committee of our center was 18.7 ± 13.4 years. The mean time between the SEEG decision and the performance of the technique was 19 ± 32.5 months.

### Surgical aspects

A total of 958 electrodes were inserted. The mean number of electrodes per patient was 14 ± 4.4 electrodes. In 38 patients the electrodes were placed in one brain hemisphere (54%) while in the remaining 33 patients were placed bilaterally (46%). The mean duration of the surgical procedure was 200 ± 55.8 min (3 h approx.)

**Fig. 2:**
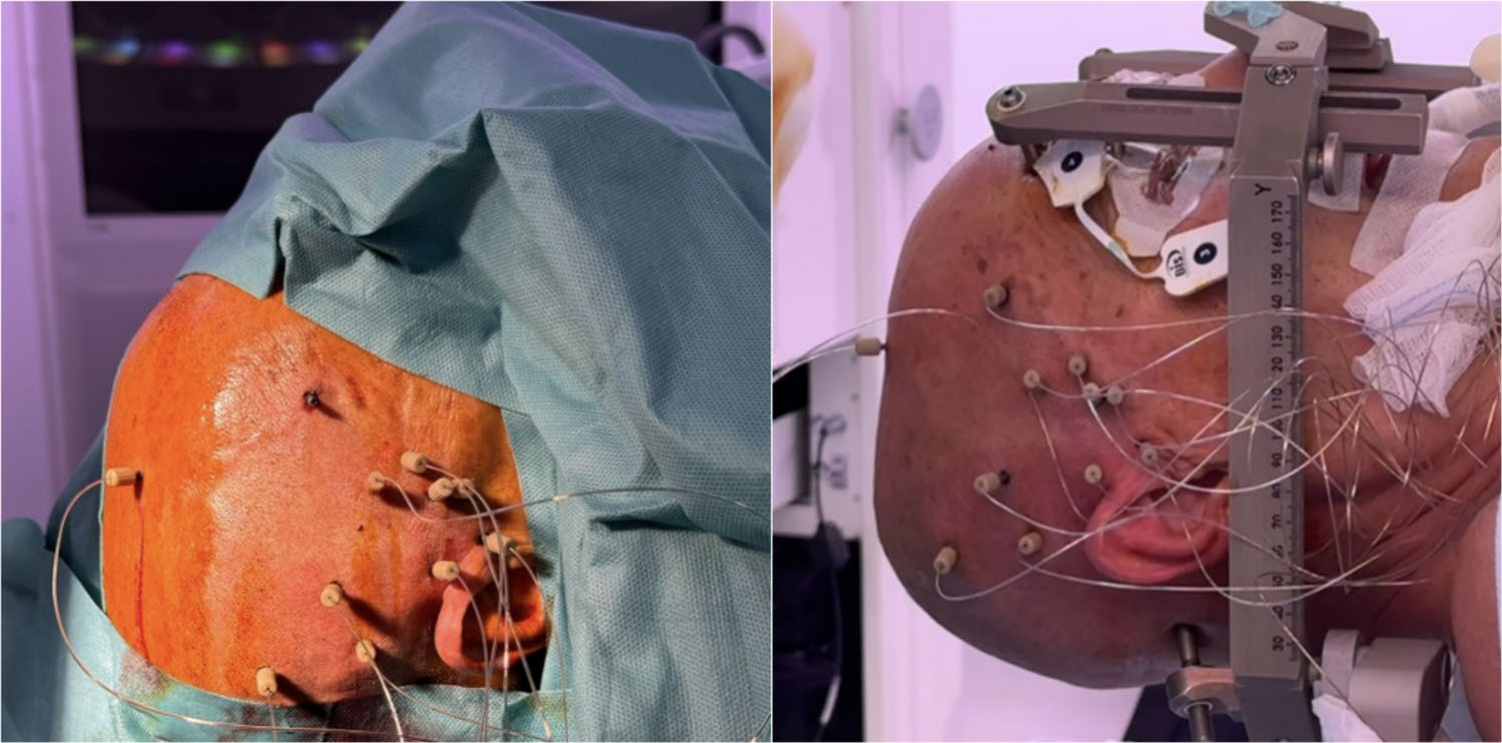
Patient undergoing SEEG monitoring with depth electrodes anchored by bolts

The mean duration of the monitoring was 13.7 ± 4.6 days, resulting in a mean hospital stay of 16.8 ± 5 days (Fig. 2).

### Complications

SEEG-related events were initially identified in 14 patients (20%). However, most of these corresponded to minor radiological findings without clinical consequences, mainly small postoperative hemorrhagic changes detected on routine CT imaging. Specifically, 8 patients (11%) had small intracerebral hemorrhages and 3 (4%) had limited subarachnoid hemorrhage, none of which produced symptoms or required medical or surgical treatment. Two patients (3%) developed infection. Only one patient (1%) experienced a clinically significant intracerebral hemorrhage requiring surgical evacuation, which resulted in a permanent neurological deficit. No deaths occurred in this series.

### Thermocoagulation (RF-TC)

RF-TC was performed after an electrical stimulation study on 37 patients (52%) in our series with the following indication: electrodes located in the EZ evaluated during the monitoring to ensure that these areas were not located in eloquent brain regions.

Among the 37 patients who underwent RF-TC, 27 (73%) experienced some degree of seizure improvemen**t** at 12 months. However, according to Engel classification, only 41% achieved favorable outcomes (Engel classes I–II), whereas 19% had Engel class III outcomes and 41% remained in Engel class IV. These findings suggest that RF-TC may provide meaningful symptomatic benefit in selected patients, although its effect is more appropriately interpreted as adjunctive or palliative rather than definitively curative.

Only 5 patients (14%) developed transient complications like a hemiparesis (2 patients), aphasia/dysphasia (2 patients) and dysnomia (1 patient).

Beyond its direct therapeutic effect, RF-TC played a relevant role in guiding subsequent treatment strategies. In patients who experienced meaningful seizure reduction following RF-TC, conservative management or delayed surgical decision-making was often favored.

Conversely, in patients with limited or no response to RF-TC, resective surgery was more frequently pursued after multidisciplinary reassessment.

This dynamic use of RF-TC suggests its value not only as a palliative intervention, but also as a functional tool within a staged and adaptive decision-making process.

### Hypothesis

The hypothesis was confirmed in 60 patients (85%), understanding the SEEG as a useful tool for the accurate diagnosis in drug-resistant epilepsy with diagnostic gaps when using general paraclinical tests.

A total of 37 patients ultimately underwent resective epilepsy surgery. Among them, 19 had previously received RF-TC as an initial therapeutic approach. In these cases, RF-TC did not lead to clinically meaningful seizure improvement, and resective surgery was subsequently indicated following multidisciplinary evaluation.

Postoperative seizure outcomes in the 37 surgically treated patients were evaluated at 3, 6, and 12 months using the Engel classification. At 3 months of follow-up, Engel class I outcomes were observed in 57% of patients, while 19% were classified as Engel class II, resulting in favorable seizure control (Engel classes I–II) in 76% of cases. At 6 months, Engel class I outcomes decreased to 51%, with Engel class II remaining stable at 19%, yielding favorable outcomes in 70% of patients. At 12 months, Engel class I outcomes were observed in 49% of patients, while Engel class II increased slightly to 22%, maintaining favorable seizure control in 71% of the cohort. Across follow-up intervals, Engel class III outcomes ranged from 16 to 22%, whereas Engel class IV outcomes increased modestly over time, reaching 14% at 12 months (Table 2).

**Table 2:** All percentages are calculated based on the total cohort (*n* = 37). Seizure outcomes were classified according to the Engel postoperative outcome scale at 3, 6 and 12 month follow-up. Favorable outcomes were defined as Engel classes I–II

Postoperative Seizure Outcomes at 3, 6, and 12 Month Follow-up According to the Engel Classification			
Month	Engel Class	*n*	%
3	I	21	57
	II	7	19
	III	6	16
	IV	3	8
6	I	19	51
	II	7	19
	III	8	22
	IV	3	8
12	I	18	49
	II	8	22
	III	6	16
	IV	5	14

34 patients were not candidates for resective epilepsy surgery. The main reasons included rejection of surgery by the patient (26%), multifocal epilepsy (24%), partial seizure improvement achieved with pharmacological adjustment combined with effective RF-TC (21%), high surgical risk (defined as the EZ located within eloquent brain areas) (18%), and absence of disabling seizures at the time of evaluation (12%).

Among the patients excluded from resective surgery due to multifocal epilepsy (*n* = 8), alternative neuromodulation-based surgical treatments were offered. Of these, 50% underwent deep brain stimulation of the anterior nucleus of the thalamus, 13% received deep brain stimulation of the centromedian nucleus, and 38% were treated with vagus nerve stimulation (Table 3).

**Table 3:** All percentages are calculated based on the total number of patients who were not candidates for resective surgery (*n* = 34). RF-TC: radiofrequency thermocoagulation

Reasons for Not Undergoing Resective Epilepsy Surgery		
Characteristic	*n*	%
Rejection of surgery	9	26
Multifocality	8	24
Partial seizure improvement with pharmacological adjustment + RF-TC	7	21
High surgical risk	6	18
Absence of disabling seizures	4	12

High surgical risk refers to cases in which the EZ involved eloquent cortical areas

### SEEG-guided therapeutic decision pathways

SEEG findings enabled classification of patients into distinct therapeutic pathways, directly influencing clinical decision-making in all cases.

Following SEEG evaluation, 37 patients (52%) were considered suitable candidates for resective epilepsy surgery. In contrast, 34 patients (48%) were not offered resective treatment based on SEEG findings and multidisciplinary evaluation.

Importantly, SEEG contributed to the avoidance of potentially ineffective or high-risk surgical interventions in these patients. The main reasons for exclusion included multifocal epilepsy (24%), localization of the epileptogenic zone within eloquent cortex (18%), and insufficiently disabling seizures (12%). Additionally, 26% of patients declined surgery after SEEG-informed discussion.

These findings highlight the role of SEEG not only in selecting surgical candidates, but also as a critical gatekeeping tool in preventing inappropriate surgical indications.

## Discussion

This study expands the role of stereoelectroencephalography (SEEG) beyond its traditional function as a diagnostic tool for epileptogenic zone localization, positioning it as a central platform for structured therapeutic decision-making in drug-resistant epilepsy. While most previous series have focused primarily on surgical outcomes or procedural safety, our findings emphasize the value of SEEG in dynamically guiding patient-specific treatment pathways, including resective surgery, minimally invasive interventions such as radiofrequency thermocoagulation (RF-TC), neuromodulation strategies, or conservative management.

In this series, nearly half of the patients did not proceed to resective surgery following SEEG evaluation, underscoring its importance in identifying cases where surgery may be ineffective or associated with unacceptable functional risk.

This highlights a critical but often underreported function of SEEG: its role as a gatekeeper in the surgical decision-making process. By preventing unnecessary or inappropriate resections, SEEG contributes not only to improved outcomes but also to safer patient selection.

A key contribution of this study is the formalization of SEEG as an operational decision-making framework. By structuring SEEG findings into sequential steps—hypothesis validation, epileptogenic zone characterization, functional testing through RF-TC, and final therapeutic allocation—we provide a reproducible model that may facilitate clinical application across centers. This approach moves beyond descriptive interpretation and supports a standardized, yet flexible, strategy for integrating invasive electrophysiology into real-world surgical decision-making.

**Fig. 3:**
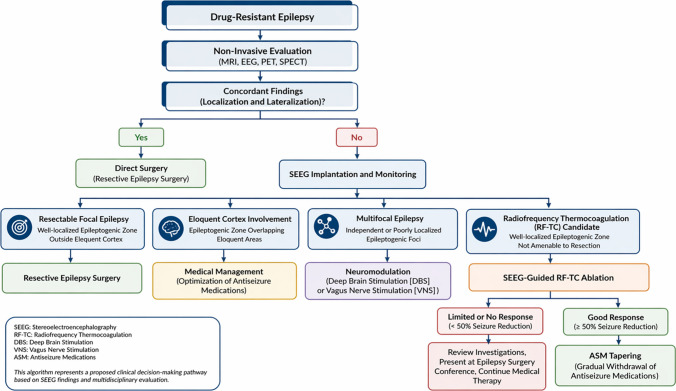
SEEG-guided clinical decision-making algorithm in drug-resistant epilepsy. SEEG findings guide patient stratification and individualized treatment selection

The proposed framework derived from our institutional experience is summarized in Fig. 3

In this context, SEEG-guided RF-TC should not be interpreted solely as a palliative intervention, but rather as an integral component of a staged therapeutic strategy. Beyond its direct clinical effect, RF-TC provides functional insight into the epileptogenic network, allowing real-time assessment of the impact of focal disruption. In our series, the differential response to RF-TC played a decisive role in subsequent management, favoring conservative or delayed approaches in responders, while supporting progression to resective surgery in non-responders. This dual diagnostic–therapeutic role reinforces the concept of SEEG as an adaptive decision-making tool rather than a static diagnostic procedure, as illustrated in Fig. 1.

Technical refinements, including the introduction of robotic assistance, have further improved accuracy and safety in SEEG procedures. Gonzalez-Martinez et al. (2016) reported high precision with robotic implantation, reducing trajectory error and potentially minimizing complication risk [13]. Similarly, Cardinale et al. (2019) highlighted how modern stereotactic platforms and imaging integration contribute to improved outcomes and workflow efficiency [5]. Our own experience mirrors this evolution, as since 2023 we transitioned from the Vertek passive arm to the Neuromate® robotic system, maintaining safety while enhancing implantation accuracy [25]. Moreover, the implementation of computer-assisted planning platforms has recently shown potential to optimize RF-TC trajectories and outcomes, particularly in pediatric epilepsy [31].

Our complication rate was 20% and consisted predominantly (86%) of minor intracerebral hemorrhages (< 5 cc), which had no clinical impact on patients and were therefore considered minor radiological findings; only one case (1%) developed an intracerebral hemorrhage requiring surgical drainage, resulting in permanent motor disability. These results are in line with large meta-analyses such as Mullin et al., who reported a pooled prevalence of 1.3% for clinically significant complications [2], and with McGovern et al., who emphasized the low risk of hemorrhage when proper planning and trajectory selection are applied [21].

Regarding RF-TC, our seizure improvement rate of 73% with seizure freedom in 14% compares favorably with recent long-term series. Lagarde et al. (2021) demonstrated that thermocoagulation can provide durable seizure reduction in carefully selected patients, with seizure freedom ranging from 10–20% [17].

Recent analyses have also identified clinical predictors of favorable outcomes in MRI-negative patients undergoing RF-TC, further supporting the role of this approach in carefully selected populations [15].

These findings support a clinically meaningful adjunctive role for RF-TC in selected patients, particularly when resective surgery is not feasible or is deferred. Although complete seizure freedom was limited, RF-TC provided worthwhile seizure reduction in a substantial proportion of patients, supporting its value as a palliative or bridge therapy within a broader individualized surgical strategy.

From a clinical perspective, this framework supports a shift from a binary surgical paradigm toward a more nuanced, patient-specific strategy. Rather than simply determining whether a patient is a candidate for resection, SEEG enables stratification across multiple therapeutic pathways, optimizing both efficacy and safety. This paradigm is particularly relevant in complex cases, including MRI-negative epilepsy, multifocal disease, or epileptogenic zones overlapping eloquent cortex, where conventional decision-making is often uncertain.

## Limitations

This study has several limitations. First, its retrospective and single-center nature may limit generalizability. Second, although all patients were consecutively included, referral bias inherent to a tertiary epilepsy center must be considered. Third, our clinical practice evolved during the 9-year study period, particularly regarding imaging quality and the adoption of robotic assistance (transitioning from the Vertek passive arm to the Neuromate® system), which may have influenced complication rates and accuracy. Fourth, although follow-up was performed in all patients, the duration varied, and longer-term data are required to assess the durability of seizure outcomes, particularly after thermocoagulation. Finally, the relatively small sample size compared to multicenter meta-analyses may partly explain the differences in infection rates observed. In addition, the identification of reliable predictors of response to RF-TC was beyond the scope of this study and warrants further investigation in larger prospective cohorts.

The patient population presented here reflects a complete series of consecutively explored patients, representative of our center’s practice over a 9-year period. Our clinical practice has evolved over this period along with other aspects of our investigation such as the acceptance of thermocoagulation and the precision of MRI studies.

Future studies should aim to validate this framework prospectively and to identify predictive factors that may further refine patient selection and optimize individualized treatment strategies.

## Conclusions

SEEG represents a robust, safe, and highly effective diagnostic modality for the presurgical evaluation of patients with complex drug-resistant epilepsy. By enabling precise delineation of the EZ, SEEG substantially enhances the accuracy of surgical decision-making and optimizes the selection of candidates for resective procedures. When integrated with tailored resective surgery, SEEG contributes to achieving excellent long-term seizure control and meaningful improvements in quality of life. Furthermore, SEEG-guided RF-TC may represent a valuable adjunctive therapeutic option in carefully selected patients, particularly when resection is not feasible or when temporary seizure reduction may inform subsequent treatment decisions. Taken together, these advances underscore SEEG not merely as a diagnostic tool but as a cornerstone in the modern multidisciplinary management of drug-resistant epilepsy —bridging innovation, precision, and therapeutic success. Ultimately, SEEG redefines the limits of presurgical evaluation and paves the way toward a future where individualized epilepsy surgery becomes not the exception, but the standard of care.

## Data Availability

No datasets were generated or analysed during the current study.

## Declarations

### AI use Declaration

No generative artificial intelligence tools were used for the generation of content, data analysis, or manuscript writing. Only minor language and grammar edits were performed under full author supervision, in compliance with the ethical publishing policies of Acta Neurochirurgica.

### Reporting guidelines

This retrospective observational cohort study has been prepared in accordance with the STROBE (Strengthening the Reporting of Observational Studies in Epidemiology) statement. A completed STROBE checklist is submitted as a supplementary file.
